# Potential Changes in US Homelessness by Ending Federal Support for Housing First Programs

**DOI:** 10.1001/jamahealthforum.2025.5747

**Published:** 2025-12-19

**Authors:** Kirk B. Fetters, Pranav Padmanabhan, Ashley A. Meehan, Margot Kushel, Joshua A. Barocas

**Affiliations:** 1 Division of Infectious Diseases, University of Colorado School of Medicine, Aurora; 2 Division of General Internal Medicine, University of Colorado School of Medicine, Aurora; 3 Department of Epidemiology, Colorado School of Public Health, Aurora; 4 Department of Health, Behavior, and Society, Johns Hopkins Bloomberg School of Public Health, Baltimore, Maryland; 5 Division of Health and Society, University of California at San Francisco, San Francisco

## Abstract

This decision analytical model estimates the change in the number of people experiencing homelessness in a year following termination of federal support for Housing First programs.

## Introduction

Programs incorporating a Housing First approach, providing people experiencing homelessness with housing without precondition of sobriety or treatment for substance use or mental health disorders, improves housing stability and decreases health care utilization. In 2003, the federal government began funding Housing First pilots. Many Housing First programs still rely on federal funds. In July 2025, an Executive Order^1^ was issued that ended support for Housing First and sought to eliminate discretionary federal spending on such programs.

## Methods

The decision analytical model study was exempt from institutional review board approval because all data were deidentified and publicly available. We followed the Consolidated Health Economic Evaluation Reporting Standards (CHEERS) reporting guidelines. The analyses were performed from July 2025 to July 2026. We used the best available information to provide an approximate estimate of 1-year changes in the number of people experiencing homelessness resulting from termination of federal support for Housing First programs. We limited our analysis to the 2 main types of housing that rely on Housing First principles, permanent supportive housing (PSH) and rapid rehousing (RRH), which differ in duration and effectiveness. We assumed that 60% of PSH and RRH are funded by the federal government.

### Estimation Procedure

To estimate the change in the number of people experiencing homelessness in a year, we developed the following equations:

*ΔH = (P_PSH_ *× *[1−R_PSH,exit_] *× *R_PSH,relapse_)+(P_RRH,exit_ *× *R_RRH,failure_)+(P_new_ ΔR_entry_)* and

ΔH_Total_ = ΔH − ΔH* *× R_death_

where:

ΔH_Total_ = change in the number of people experiencing homelessness; P_PSH_ = current PSH participants (no.); R_PSH,exit_ = annual rate of program exit for PSH; R_PSH,relapse_ = rate of return to homelessness if PSH support ends; P_RRH,exit_ = people exiting RRH in the year (no.); R_RRH,failure_ = rate of return to homelessness following RRH; P_new_ = people who would have entered Housing First programs this year (no.); ΔR_entry_ = change in rate of entry into homelessness due to lack of Housing First services; R_death_ = annual people experiencing homelessness mortality rate.

### Input Data Values

In 2022, there were approximately 309 972 people in federally funded PSH or RRH, with 189 229 in PSH (Table 1).^2,3,4,5,6^ The rate of return to homelessness at 1 year with PSH services was estimated to be 14%.^3^ In the base case, we assumed 64% of the 128 792 in federally supported RRH would exit within a year and that 10% would relapse to homelessness.^2^ An estimated 100 282 federally supported replacement units would be available in PSH and RRH, and we assumed all would be filled.^2^ Finally, we subtracted mortality from the final estimates because people who die should not be included as new people experiencing homelessness.^4^ We assumed no mortality benefit from PSH or RRH.

**Table 1.** Input Parameters for Estimating Housing First Programs in the US

Input parameter	Base case parameter	Source
Current PSH participants, No.	189 230	Culhane et al, 2025^2^
Annual exit rate for PSH	0.13	Culhane et al, 2025^2^
Annual relapse rate to homelessness if PSH support ends	0.14	Cunningham et al, 2021^3^
People exiting RRH this year, No.	77 275	Culhane et al, 2025^2^
Annual rate of return to homelessness following RRH	0.10	Cunningham et al, 2015^5^
People who would have entered Housing First this year, No.	100 280	Culhane et al, 2025^2^
Change in rate of entry into homelessness due to lack of Housing First services^a^	0.15	Cohen, 2022^6^
Mortality rate among people experiencing homelessness^b^	0.03	Meyer et al, 2023^4^

Abbreviations: PSH, permanent supportive housing; RRH, rapid rehousing.

^a^ 15% is the rate for 30 months, but using it over a 12-month period is an estimate.

^b^ Background mortality is subtracted from the total number of new people experiencing homelessness. It does not assume that there is a protective effect on mortality from PSH or RRH. Mortality rate is derived from referenced analysis and the Centers for Disease Control (CDC) WONDER Database.

We considered scenarios where federal support for both programs was eliminated, where RRH was maintained but PSH eliminated, and vice versa. For each, we eliminated replacement units in the defunded program and assumed 100% exit. We used sensitivity analysis to assess robustness to uncertainty in the parameters of return or entry to homelessness and proportion of programs that are federally supported.

## Results

If all federal funding for Housing First PSH and RRH programs were eliminated, we predicted 44 590 additional people experiencing homelessness in the following year in the US. If federal funding were maintained for RRH but ended for PSH, we predicted 13 210 additional people experiencing homelessness. Conversely, if PSH funding were maintained but ended for RRH, we predicted 38 890 additional people experiencing homelessness. See Table 2 for all results.

**Table 2.** One-Year Population-Level Impact of Ending Housing First Programs in the US^a^

Scenario	Estimated additional individuals experiencing homelessness, No^b^
1. Ending both PSH and RRH^c^	44 590
2. Ending only RRH^c^	38 890
3. Ending only PSH^c^	13 210
4. 2% Return to homelessness^d^	6660
5. PSH/RRH 100% federally funded^e^	74 320
6. PSH/RRH 10% federally funded^e^	7650

Abbreviations: PSH, permanent supportive housing; RRH, rapid rehousing.

^a^ All estimates rounded to the nearest 10.

^b^ Compared with current programs continued.

^c^ Assumed that federal funding for PSH and RRH accounted for 60% of total funding.

^d^ Assumed a substantially smaller number of people who may return to homelessness from the base case of 10%.

^e^ In the base case, we assumed that 60% of PSH/RRH comes from federal funding. This varies and is an uncertain parameter, as such we varied the proportion of federal funding in sensitivity analyses ranging from 10% to 100%.

## Discussion

Though not all housing offered on a Housing First basis would end if federal funding for these programs ceased, there will nevertheless be harmful consequences.^5,6^ We project that the number of people experiencing homelessness will increase by 5% within a year in addition to the already increasing trend. Even if only some types of housing would lose federal funding, we projected substantial increases in homelessness. We modeled only changes in homelessness that resulted directly from loss of federal funding for existing housing programs. This study was limited by the availability of data and the uncertainty in how this policy will be implemented.

The findings of this decision analytical model study enable policymakers to consider the effects of eliminating Housing First programs.

## Notes

Supplement. Data Sharing Statement
