# Supplementary material for: Potential Changes in US Homelessness by Ending Federal Support for Housing First Programs
Source: JAMA Health Forum. 2025 Dec 19;6(12):e255747. doi: 10.1001/jamahealthforum.2025.5747 (PMC12717612; doi:10.1001/jamahealthforum.2025.5747)
Supplement: Supplement. — Data Sharing Statement [file jamahealthforum-e255747-s001.pdf]

Data Sharing Statement

Fetters. Potential Changes in US Homelessness by Ending Federal Support for Housing First Programs. *JAMA Health Forum*. Published December 19, 2025.
doi:10.1001/jamahealthforum.2025.5747

Data
Data available: All data are publicly available.

Additional Information
Explanation for why data not available: All data to populate this model was procured from published literature.
